# B-cell epitope mapping and characterization of antibody responses to recombinant PvRipr in malaria-exposed individuals

**DOI:** 10.3389/fimmu.2025.1710869

**Published:** 2026-01-28

**Authors:** Isabela Ferreira Soares, Cinthia Magalhães Rodolphi, Ana Luiza Carneiro Alencar, Ada da Silva Matos, Rodrigo Nunes Rodrigues-da-Silva, Barbara de Oliveira Baptista, Rodrigo Medeiros Martorano, Hugo Amorim dos Santos de Souza, Evelyn Kety Pratt Riccio, Jenifer Peixoto de Barros, Paulo Renato Rivas Totino, Cláudio Tadeu Daniel-Ribeiro, Lilian Rose Pratt-Riccio, Josué da Costa Lima-Junior

**Affiliations:** 1 Laboratório de Imunoparasitologia, Instituto Oswaldo Cruz (IOC), Fundação Oswaldo Cruz (Fiocruz), Rio de Janeiro, RJ, Brazil; 2 Laboratório de Hantaviroses e Rickettsioses, Instituto Oswaldo Cruz (IOC), Fundação Oswaldo Cruz (Fiocruz), Rio de Janeiro, RJ, Brazil; 3 Laboratório de Pesquisa em Malária, Instituto Oswaldo Cruz (IOC), Fundação Oswaldo Cruz (Fiocruz), Rio de Janeiro, RJ, Brazil; 4 Laboratório de Doenças infecciosas na Amazônia Ocidental, Universidade Federal do Acre (UFAC), Cruzeiro do Sul, AC, Brazil; 5 Centro de Pesquisa, Diagnóstico e Treinamento em Malária (CPD-MAL), Fundação Oswaldo Cruz (Fiocruz), Rio de Janeiro, RJ, Brazil

**Keywords:** B-cell epitopes, malaria, *P. vivax*, Ripr, vaccine candidate

## Abstract

**Introduction:**

Malaria caused by *P. vivax* continues to be a serious public health problem, especially in countries like Brazil where *P. vivax* accounts for more than 80% of diagnosed cases. Since this plasmodial species is characterized as one of the most difficult to eliminate, the development of a specific vaccine for *P. vivax* may be an essential tool for effective control of the disease. The protein Ripr has been described in *P. falciparum* as an essential part of the erythrocyte invasion complex. Given the limited number of *P. vivax* vaccine antigens currently under investigation, this study aimed to characterize, the naturally acquired humoral immune response to Ripr protein of *P. vivax*.

**Methods:**

ELISA assays were performed using plasma samples from individuals naturally exposed to malaria in the Brazilian Amazon in order to determine levels of IgM, IgG and IgG subclasses against PvRipr. In addition, linear B-cell epitopes within the protein were identified.

**Results and Discussion:**

Our results demonstrated that PvRipr is naturally immunogenic, as more than 60% of the individuals presented IgM or IgG antibodies against recombinant PvRipr. The profile of IgG subclasses was also investigated and higher frequencies of seropositive individuals for IgG1 and IgG2 were observed. After *in silico* prediction, a total of four linear B cell epitopes were identified in PvRipr, from these sequences, B-PvRipr_(879-888)_ and B-PvRipr_(923-958)_ had higher frequencies of seropositive individuals and reactivity indexes in comparison to the other tested epitopes. Moreover, levels of IgG antibodies specific for these two epitopes were strongly correlated with the levels of IgG antibodies against recombinant PvRipr and especially with IgG3 antibodies, a cytophilic subclass widely cited in the protective immune response against malaria.

## 1 Introduction

*Plasmodium vivax* is one of the *Plasmodium* species capable of infecting humans, being the second in number of cases and is considered the most widely distributed geographically (1). Nearly half of the global population is at risk of contracting this parasite, with its presence reported across Asia, South and Central America, Oceania, the Middle East, and specific areas of Africa, collectively threatening an estimated 2.85 billion individuals annually. Although its highest prevalence occurs in Asia and Latin America, *P. vivax* remains one of the most common malaria-causing parasites worldwide, with annual incidence ranging between 9 and 14 million cases between 2000 and 2023 (2). Most of these infections—over 85%—were concentrated in six countries: India, Afghanistan, Pakistan, Ethiopia, Papua New Guinea, and Indonesia. Globally, *P. vivax* is estimated to cause 3.5% of malaria cases, a proportion that rises to roughly half of all cases when sub-Saharan Africa is excluded (2). In the Americas, *P. vivax* is the leading cause of malaria, contrasting with the predominance of *P. falciparum* in Africa. This parasite poses a major barrier to global malaria elimination efforts due to biological features that confer marked resilience and adaptability, such as its capacity to form dormant liver stages (hypnozoites) and to infect individuals at lower parasite densities (3, 4). In addition to its epidemiological impact, *P. vivax* imposes a substantial socio-economic burden, and reports of severe disease manifestations and mortality associated with this species have become increasingly frequent. Although research and control initiatives over the past three decades have focused predominantly on *P. falciparum*, recent strategies have highlighted the urgent need to expand vaccine and drug development efforts against *P. vivax*. To date, there are only two blood stage vaccines for *P. vivax* (both based on the Duffy Binding Protein - DBP) in clinical trials in comparison to 13 blood stage vaccines for *P. falciparum* (5). This huge difference in the numbers of vaccines and antigens in clinical trials against these two plasmodial species only reinforces the importance of investigating and characterizing new blood-stage vaccine candidates for *P. vivax.*

This renewed attention has been driven by advances in genomic technologies enabling comprehensive analysis of the *P. vivax* genome, together with global commitments to malaria eradication, which collectively highlight the importance of addressing *P. vivax* as a critical public health challenge. Consequently, a wide array of antigens expressed during the asexual blood stages of *P. vivax* have been identified and immunologically characterized. Among these, proteins located on the merozoite surface or within apical organelles have received particular interest as vaccine candidates. Prominent classical examples include the *P. vivax* merozoite surface protein 1 (PvMSP-1), the PvMSP-3 family, PvMSP-9, reticulocyte-binding protein 1 (PvRBP-1), apical membrane antigen 1 (PvAMA-1), and Duffy binding protein (PvDBP). Recently other antigens were also included in this list, such as PvCyRPA, PvGAMA and others (6, 7).

It is already well established that the naturally acquired immune response against *Plasmodium* develops slowly with age and exposure, besides, it is characterized as a complex mechanism that involves both innate and adaptive immune responses with a stage-specific profile (8). Thus, considering the existence of thousands of *Plasmodium* proteins, the combination of antigens in the form of a chimeric construct has been explored as a promising strategy in the investigation of anti-malarial vaccines, capable of inducing a broader and more efficient immune response (9–13). Within the approaches currently used in malaria vaccine studies, blood stage vaccines are composed of merozoite antigens, the stage of *Plasmodium* responsible for malaria clinical symptoms. It has already been demonstrated for *P. falciparum* that individuals who are repeatedly exposed to infection in endemic areas develop both immunity to clinical disease and blood stage infection (14, 15). In this scenario, in order to perform the appropriate selection of blood stage antigens, a deep understanding of the erythrocyte invasion process is necessary, which involves multiple interactions between key merozoite proteins (especially those present in membrane-bound organelles, such as micronemes and rhoptries) and host red blood cell receptors (16).

The Rh5 interacting protein (Ripr) was described in *P. falciparum* merozoites as a part of an essential invasion complex composed of cysteine-rich protective antigen (CyRPA), a micronemal protein, and reticulocyte binding protein homologue 5 (Rh5), secreted by the rhoptries. Ripr also localizes in the microneme, from where it migrates to the merozoite membrane surface and interacts with CyRPA and Rh5 (17, 18). No orthologue of Rh5 was identified in the genomes of any other *Plasmodium* species outside the *Laverania* subgenus, which is curious, given the apparent key role of Rh5 in *P. falciparum* erythrocyte invasion. In the case of *P. knowlesi*, it was demonstrated that PkRipr and PkCyRPA are both essential for parasite invasion and survival, however, they do not interact with each other and present independent functions. PkRipr interacts with two other proteins, the small cysteine-rich secreted protein PkCSS and the thrombospondin-related apical merozoite protein PkTRAMP (19). Considering that *P. vivax* and *P. knowlesi* are very close phylogenetically with 89% of gene orthologs between them (20), it is likely that PvRipr and PvCyRPA have also indispensable and independent functions for the success of parasite invasion. On the other hand, to date, no information is available regarding the antibody profile and subclasses induced against PvRipr and the location and characteristics of its main epitopes Furthermore, the relationship with the specific immune response against PvRipr and host exposure history and/or possible contribution to protective immunity have not yet been determined.

Therefore, considering the few blood stage antigens in clinical trials for *P. vivax*, associated with its epidemiological impact for global control of malaria, the purpose of this work was to investigate, for the first time, the naturally acquired immune response of Brazilian Amazon individuals against both recombinant PvRipr and its most promising linear B cell predicted epitopes. Together, these data will provide a better understanding of the humoral immune response against PvRipr and maybe strengthen this antigen as a potential vaccine candidate for *P. vivax* and as a possible part of a specific chimeric blood stage vaccine for *P. vivax*.

## 2 Materials and methods

### 2.1 Study area and volunteers

A cross-sectional cohort study was conducted with 300 volunteer individuals from the malaria-endemic region of Acre state in Brazil from June to August 2018. Samples and survey data were collected from all individuals. Malaria transmission in Acre state is considered high and predominantly caused by *Plasmodium vivax*, with *P. falciparum* occurring at lower frequencies. The region is primarily rural, and inhabitants are frequently exposed to forested areas, increasing the risk of malaria infection. Transmission shows seasonal variation, peaking during the rainy season from November to March. In 2018, the state of Acre reported 25,806 confirmed malaria cases, remaining one of the Brazilian states with the highest malaria burden due to continued transmission in Amazonian rural areas (21). Additionally, 10 individuals living in non-endemic areas of Rio de Janeiro were also used as the non-exposed control group in order to determine cut-offs for all experiments. The methods used in the present study were carried out in accordance with the revised Declaration of Helsinki. The experimental protocols were previously approved by the Fundação Oswaldo Cruz Ethical Committee and the National Ethical Committee of Brazil (CEP-FIOCRUZ CAAE 46084015.1.0000.5248). Written informed consent was obtained from all of the study participants.

### 2.2 Epidemiological survey

To evaluate epidemiological factors that could influence the humoral immune response against PvRipr all donors were interviewed. During the survey, participants answered questions related to personal exposure to malaria, for example, years of residence in an endemic area, the number of previous malaria episodes, time since the last malaria episode, presence/absence of symptoms, and use of malaria preventive measures, among others. All epidemiological data collected were stored in Epi-info (Centers for Disease Control and Prevention, Atlanta, GA, USA) for further analysis.

### 2.3 Blood sampling and malaria diagnosis

Plasma samples were collected, stored at -20°C, and transported to our laboratory for antibody assays. Blood samples from all donors were examined for malaria parasites with thin and thick blood smears. Parasitological evaluations were done by examination of 200 fields at 1,000× magnification under oil immersion and a research expert in malaria diagnosis examined all slides. In order to heighten the sensitivity of parasite detection, DNA was extracted from patients’ blood samples by QIAamp DNA blood midi kit (Qiagen, Germantown, MD, USA) as per the manufacturer’s instructions, and submitted to Polymerase chain reaction (PCR) using specific primers for the genus (*Plasmodium* sp.) and species (*P. vivax and P. falciparum*) as described in our previous works (13, 22). Donors positive for *P. vivax* and/or *P. falciparum* at the time of blood collection were subsequently treated using the chemotherapeutic regimen recommended by the Brazilian Ministry of Health. To ensure a robust and reliable definition of infection, only samples positive by both microscopy and PCR were considered infected, thereby reducing potential errors from single-method detection.

### 2.4 Recombinant PvRipr expression

**Figure 1:**
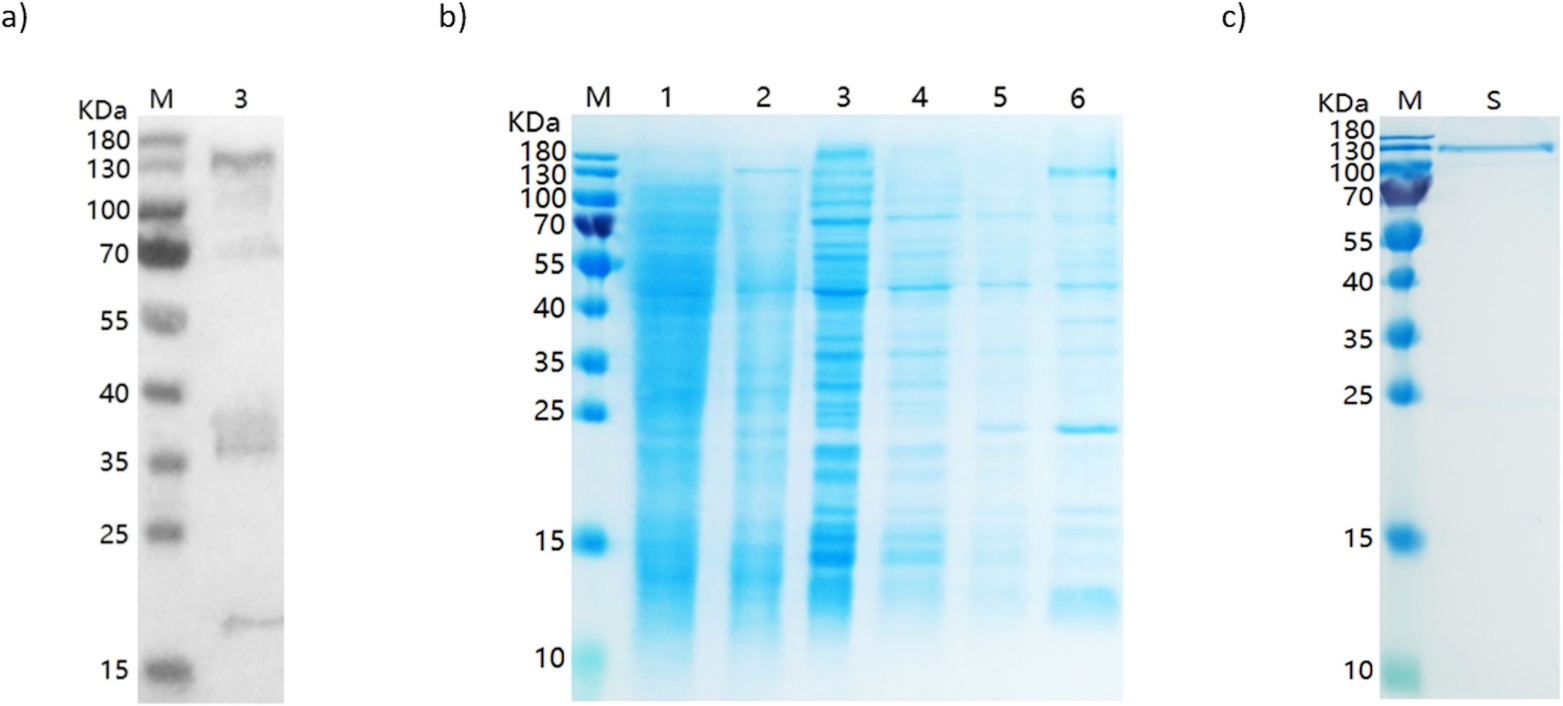
PvRipr expression and purification results. **(A)** Western blot result. Anti-His detection and ECL revelation. M: Protein Marker and 3: induced sample. **(B)** SDS-PAGE result of affinity chromatography showing the first results of expression and purification. M: Protein Marker; 1: Precipitate; 2: Supernatant; 3: Flow through; 4-5: Wash; 6: First Elution. **(C)** Final product: SDS-PAGE of the final protein expression after subsequent elutions and new dialysis step. M: Protein Marker; S: Final Sample.

The recombinant protein PvRipr was expressed in *Escherichia coli* and produced using the entire protein amino acid sequence of *P. vivax* Ripr (PVP01_0816800), containing 1074 amino acids. The plasmid containing the target gene was transformed into *E. coli* BL21(DE3) competent cells. Transformed colonies were grown in LB medium supplemented with ampicillin until reaching mid-logarithmic phase. Expression was induced by the addition of 0.5 mM isopropyl-β-D-thiogalactopyranoside (IPTG), followed by incubation at 37°C for 4 h with shaking. To optimize expression and assess protein solubility, additional cultures were induced with 0.2 mM or 1.0 mM IPTG under two different conditions: (i) 37°C for 4 h and (ii) 15°C for 16 h. Cells were harvested by centrifugation and resuspended in lysis buffer (50 mM Tris-HCl, 300 mM NaCl, pH 8.0). Cellular disruption was performed by ultrasonication (Φ3 probe, 3 s on/6 s off cycles, total of 5 min). The lysates were centrifuged to separate soluble and insoluble fractions, and expression levels were analyzed by SDS-PAGE and Western blot using an HRP-conjugated anti-His antibody (**Figure 1a**). The recombinant protein was detected but was predominantly insoluble under the tested conditions. For purification, inclusion bodies were solubilized in 50 mM Tris, 300 mM NaCl, and 8 M urea (pH 8.0). The lysate was subjected to immobilized metal affinity chromatography (IMAC) using Ni-NTA 6FF resin. After equilibration in lysis buffer, the resin was sequentially washed with the same buffer supplemented with 20 mM and 50 mM imidazole to remove non-specific proteins. The bound recombinant protein was eluted using buffer containing 500 mM imidazole. Eluted fractions containing the target protein were pooled and further purified by ion-exchange chromatography on Q Sepharose FF resin equilibrated with 50 mM Tris and 8 M urea (pH 8.0). Proteins were eluted with a linear gradient of NaCl (0–1 M). Fractions containing the protein of interest were combined and dialyzed into storage buffer (50 mM Tris, 500 mM NaCl, 2 mM DTT, pH 8.0) (**Figures 1b, c**).

### 2.5 PvRipr B-cell linear epitope prediction

For the prediction of B cell epitopes, we have explored the protein’s data from the Uniprot database (https://www.uniprot.org, ID: A0A1G4GWB5) and its respective tridimensional structure modeled by Aplhafold. The prediction of linear B cell epitopes was performed using eight different prediction algorithms: BCPreds (http://ailab-projects2.ist.psu.edu/bcpred/, accessed on October 22, 2022), BepiPred 1.0, BepiPred 2.0 and EMINI Surface Accessibility Prediction from Immune Epitope Database - IEDB (http://tools.iedb.org/bcell/, accessed on October 22, 2022), ABCPred (https://webs.iiitd.edu.in/raghava/abcpred/, accessed on October 22, 2022), Ellipro (http://tools.iedb.org/ellipro/, accessed on October 22, 2022), LBtope (https://webs.iiitd.edu.in/raghava/lbtope/protein.php, accessed on October 22, 2022) and SVMtrip (http://sysbio.unl.edu/SVMTriP/, accessed on October 22, 2022).

BCPreds is a method for predicting linear B cell epitopes using a support machine vector learning method, using as a threshold 75%. BepiPred 1.0, a server based on a combination of Markov model and a propensity scale method, considering scores above 0.35. BepiPred 2.0, a server that uses a Random Forest algorithm trained on epitopes and non-epitope amino acids determined from crystal structures, using as a threshold 0.5. EMINI Surface Accessibility Prediction calculates the surface accessibility of the linear epitopes and was used with a threshold of 1.0. ABCPred is a server with an artificial network based on the BCIPEP database that was used with scores higher than 0.84. Ellipro identifies epitopes in a determined sequence using a combination of methods such as Thornton’s method, a residue clustering algorithm, a modeler program, and Jmol viewer, the considered threshold was 0.6. LBtope, which combines several models using various techniques on a large dataset of B cell epitopes and non-epitopes, uses as threshold 0.7. SVMtrip combines Support Vector Machine method with Tri-peptide similarity and Propensity scores, considered values were above 0.4. After prediction, only the sequences predicted totally or partially in at least 5 of the 8 prediction tools used were selected for the study.

After prediction, the epitope location in the 3D structure of PvRipr was determined. Once full-length PvRipr 3D structure available in the Uniprot database did not provide sufficient structural resolution to accurately map the predicted epitopes, we tried to create novel 3D structures using Robetta (https://robetta.bakerlab.org/submit.php). Knowing that the size and conformation of PvRipr are challenging issues for protein modeling, both the entire FASTA of PvRipr and its C-terminal region (from amino acids 604 to 1074) were used. Only the C-terminal region of PvRipr (Ct-PvRipr) was capable of being modeled using the aforementioned tool, therefore, the Ct-PvRipr 3D structure was used, since all predicted epitopes were inserted in this region. Although AlphaFold predictions for PvRipr were available, they included low-confidence and incomplete segments due to the large size and complexity of the protein, and were therefore not suitable for accurate epitope mapping.

### 2.6 Peptide synthesis

After prediction, four sequences were identified as potentially antigenic linear B-cell epitopes (B-PvRipr_(698-725)_ - CVCSDSSQIEEGHLCVPKNKCKRKEYQQ, B-PvRipr_(751-771)_ – FKRNERGICIPVDYCKNVTCK, B-PvRipr_(879-888)_ - KSRSGDSPEG and B-PvRipr_(923-958)_ – GENYRPRGKDSPTGQAVKRGEATKRGDAGQPGQAHS) and were chemically synthesized by the company GenOne Biotechnologies, Brazil. Analytical chromatography of the peptides demonstrated a purity of approximately > 95%, and mass spectrometry analysis of the peptides indicated their expected mass.

### 2.7 Antibody assays

Anti-PvRipr-specific antibodies were evaluated using enzyme-linked immunosorbent assay (ELISA). Briefly, MaxiSorp 96-well plates (Nunc, Rochester, NY, USA) were coated with PBS containing 2 µg/ml of recombinant PvRipr. After overnight incubation at 4°C, the plates were washed with PBS-Tween 0.05% and blocked with PBS-Tween 0.05% containing 5% of non-fat dry milk (NFDM) for 1 h at 37°C. Individual plasma samples were diluted 1:100 in PBS-Tween-(NFDM) 2.5%, and 100 µl of the diluted samples were added to each well in duplicate. After 1 h at 37°C and three washings with PBS-Tween 0.05%, bound antibodies were detected with peroxidase-conjugated goat anti-human IgM or IgG (Sigma, St. Louis), depending on the type of ELISA test performed, followed by the addition of 3,3′,5,5′-tetramethylbenzidine (TMB) and 15 minutes later, stop solution composed of 0.2M sulfuric acid (Scienco Biotech). Optical density was identified at 450 nm using a SpectraMax 250 ELISA reader (Molecular Devices, Sunnyvale, CA, USA). The results for total IgM and IgG were expressed as reactivity indexes (RIs), which were calculated by the mean optical density of an individual’s tested sample divided by the mean optical density of 10 non-exposed control individuals’ samples plus 3 standard deviations. Subjects were scored as seropositive individuals for PvRipr if the RI’s of IgM and IgG identified against the recombinant protein were higher than 1. Additionally, the RIs of IgG subclasses were evaluated in the seropositive individuals’ group by the same method, using peroxidase-conjugated goat anti-human IgG1, IgG2, IgG3, and IgG4 (Sigma, St. Louis). For B-cell epitope mapping, ELISA assays were performed as previously described using 5 µg/ml of each synthetic peptide in PBS to identify levels of IgG antibodies against all four predicted B cell epitopes. In this experiment, plasma samples were incubated for 2 h at 37°C.

Finally, a depletion ELISA was performed with seropositive individuals for both the recombinant protein and the most promising peptides. The assay followed the same protocol described above, except that, after a 2h incubation on peptide coated plates (5 µg/ml), samples were transferred to the recombinant protein coated plate for an additional hour of incubation before the addition of the anti-human IgG antibodies.

### 2.8 Statistical analysis

Statistical analyses were done in GraphPad Prism 8.0 for Windows (GraphPad Software, Inc.). Normality tests were done in all variables using the D’Agostino & Pearson test. Kruskal-Wallis and Dunn’s multiple comparison were used for analysis of RIs of IgM, IgG and its subclasses against recombinant PvRipr and RIs of IgG specific to B cell epitopes. Additionally, chi-square test was performed to assess the differences in proportions of seropositive individuals for IgM, IgG and IgG subclasses against PvRipr and its B cell epitopes. Correlations between immune response and epidemiological parameters were evaluated by the Spearman rank test. All p-values < 0.05 were considered significant.

### 2.9 Ethics approval and consent to participate

Written consent was obtained for the use of all collected samples. Obtained survey data was also in accordance with the revised Declaration of Helsinki. Both collection and consent protocols were under approval of Fundação Oswaldo Cruz Ethical Committee and the National Ethical Committee of Brazil (CEP-FIOCRUZ CAAE 46084015.1.0000.5248).

## 3 Results

### 3.1 Clinical and epidemiological profile of the population naturally exposed to malaria

The studied population consisted of 300 individuals naturally exposed to malaria from Acre state. This population was composed of 151 men (50.3%) and 149 women (49.7%), with medians of 32 years of age (interquartile range – IQR 21-48) and of 31 years living in endemic areas (IQR 20-46). The population has a median of 8 previous episodes of malaria (IQR 3-18) and among the group of individuals who reported a previous history of the disease, according to reports collected from patients, 249 (83%) had already been infected with *P. vivax*. Besides, 82 (27.3%) individuals were diagnosed with *P. vivax* during sample collection (microscopy and PCR), all these data are demonstrated in **Table 1**.

**Table 1:** Epidemiological data of the studied population.

Epidemiological data	(N = 300)
Gender N (%)	
Male	151 (50.3%)
Female	149 (49.7%)
Exposure factors median (IQR)	
A.P.I	61,4
Age (years)	32 (21-48)
Time of residence in endemic area (years)	31 (20-46)
Time of residence in the present address (years)	21.5 (12.25-38)
Months since the last malaria episode	9.5 (2-24)
Number of malaria episodes in the last year	0.5 (0-2)
Number of previous malaria episodes	8 (3-18)
Previously infecting species	N (%)
*P. vivax*	58 (19.3%)
*P. falciparum*	21 (7%)
*P. vivax and P. falciparum*	191 (63.7%)
No *Plasmodium* infections	7 (2.3%)
Not reported	23 (7.7%)
Diagnosis (Thick smear and PCR)	N (%)
*P. vivax*	81 (27%)
*P. falciparum*	18 (6%)
Mixed	1 (0.3%)
Negative	200 (66.7%)

The collected epidemiological data were submitted to D’Agostino & Pearson test, once the result of the test indicated that the Gaussian distribution was not normal, only nonparametric statistical tests were performed with these data.

### 3.2 Reactivity of IgM and IgG antibodies from naturally exposed individuals against recombinant PvRipr

**Figure 2:**
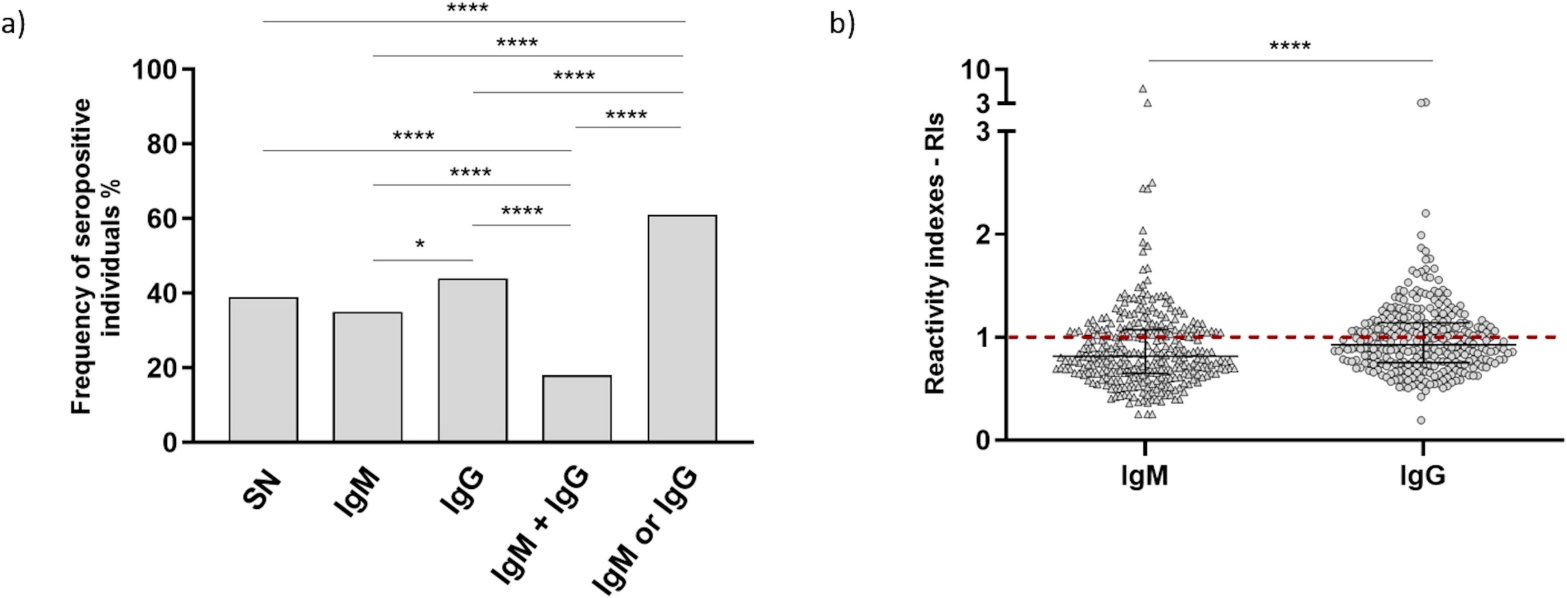
Frequencies of IgM and IgG seropositive individuals for recombinant PvRipr. The p values are <0.0001 for **** and 0.0049 for *. SN = seronegative individuals. **(B)** RIs of IgM and IgG antibodies against recombinant PvRipr. Triangles represent RIs of each individual for IgM antibodies and circles for IgG antibodies. The p value is <0.0001 (****). Asterisks above bars that are not connected by capped lines indicate that the p value applies to all groups.

The prevalence of naturally acquired antibodies specific to the PvRipr recombinant protein was determined with plasma of all studied individuals. The obtained results demonstrated that 184 individuals (61.3%) presented IgM or IgG antibodies against the protein in comparison to seronegative individuals (n=116 – 38.7%). This difference was statistically significant (Fisher’s exact test, p < 0.0001). Among seropositive individuals, 106 were considered seropositive for IgM antibodies (35.3%) and 131 were seropositive for IgG antibodies (43.7%) (Fisher’s exact test, p=0.0449). Besides, 55 individuals presented both IgM and IgG antibodies against PvRipr (18.3%) (Fisher’s exact test, p<0.0001), as demonstrated in **Figure 2a**. In relation to the reactivity indexes (RIs) identified, IgM antibodies ranged from 0.253 to 6.119 with a median of 0.817. For IgG antibodies, higher RIs were found in comparison to IgM and ranged from 0.194 to 3.271 with a median of 0.927, with Mann-Whitney test, p<0.0001 (**Figure 2b**). Among all individuals seronegative for anti-PvRipr IgG antibodies (n = 169), 58 (34%) were infected at the time of sample collection: 11 with *P. falciparum*, 46 with *P. vivax*, and 1 with a mixed infection (*P. falciparum* and *P. vivax*). All collected epidemiological parameters were compared between seropositive and seronegative individuals for IgM and IgG antibodies, however, no statistical differences were found.

### 3.3 Profile of IgG subclasses induced against recombinant PvRipr

**Figure 3:**
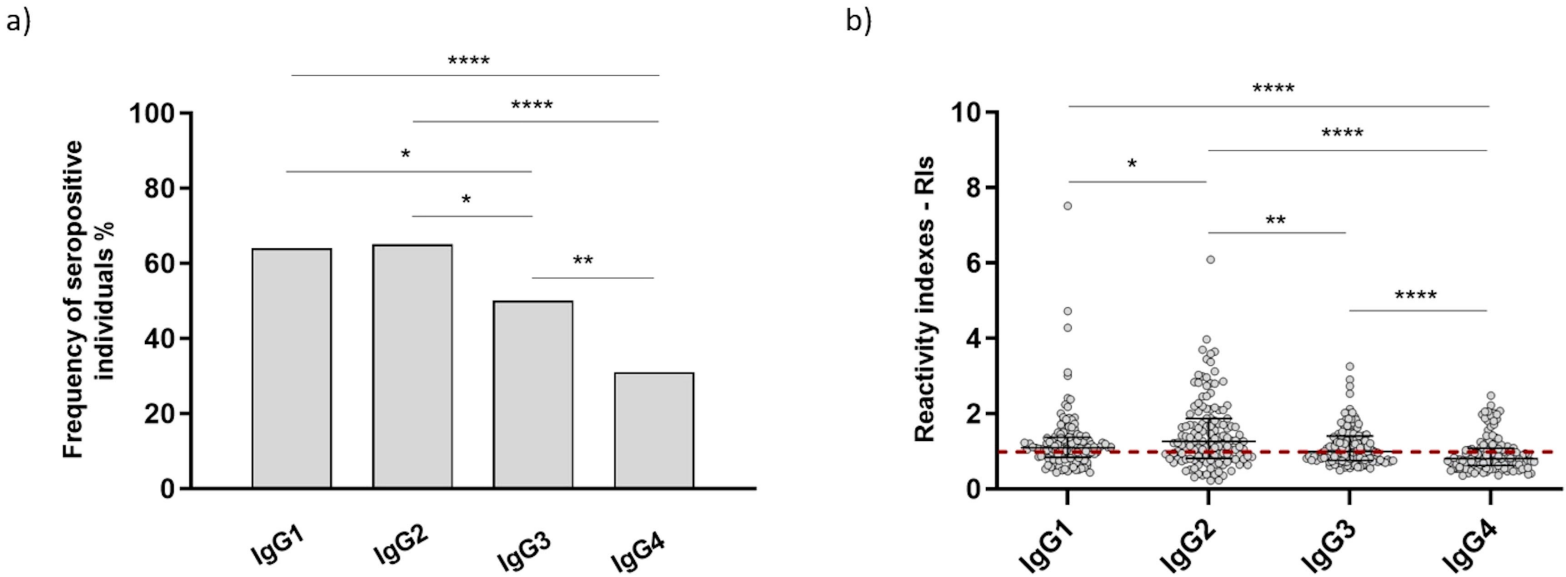
**(A)** Frequencies of seropositive individuals for IgG subclasses against recombinant PvRipr. The p values are IgG1 vs IgG3 – p=0.0245 (*), IgG2 vs IgG3 – p=0.0175 (*), p=0.0037 (**) and p<0.0001 (****) **(B)** RIs of IgG subclasses against recombinant PvRipr. The p values are p=0.0403 (*), p=0.0036 (**) and p<0.0001 (****). Asterisks above bars that are not connected by capped lines indicate that the p value applies to all groups.

All plasma samples of IgG seropositive individuals (n=131) were tested in ELISA assays for determination of the profile of IgG subclasses. From these, 84 individuals were seropositive for IgG1 (64.1%), 85 were seropositive for IgG2 (64.9%), 65 were seropositive for IgG3 (49.6%), and 41 were seropositive for IgG4 (31.3%). Frequencies of seropositive individuals for IgG1 and IgG2 were higher in comparison to IgG3 (Fisher’s exact test, IgG1 vs IgG3 – p=0.0245/IgG2 vs IgG3 – p=0.0175) and IgG4 (Fisher’s exact test, p<0.0001 for both IgG1 and IgG2). The percentage of seropositive individuals for IgG3 antibodies was also higher in comparison to IgG4 (Fisher’s exact test, p=0.0037), as demonstrated in **Figure 3a**. RIs identified in the IgG seropositive group ranged from 0.435 to 7.515 with a median of 1.093 for IgG1 antibodies, 0.222 to 6.084 and median of 1.263 for IgG2, 0.492 to 3.254 and median of 0.999 for IgG3, and 0.352 to 2.478 with a median of 0.807 for IgG4. Statistical differences were observed for RIs, since IgG2 antibodies presented higher RIs in comparison to IgG1 (p=0.0403), IgG3 (p=0.0036), and IgG4 (p<0.0001) (**Figure 3b**; all these p values were obtained using the Mann-Whitney test). From the 131 tested individuals, only 3 (2.3%) did not respond to any of the tested subclasses.

### 3.4 Prediction of B cell linear epitopes in PvRipr

**Figure 4:**
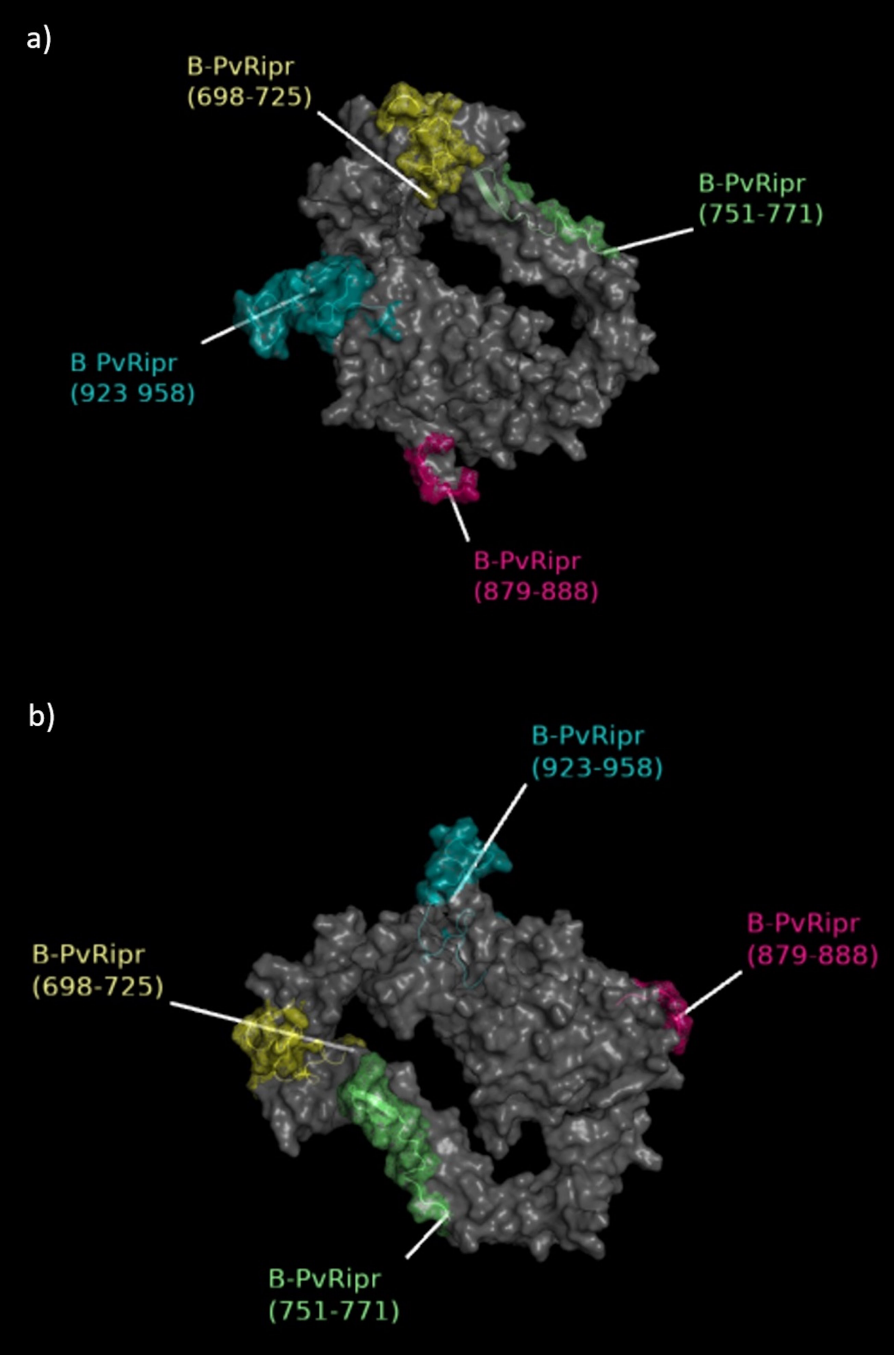
Location of predicted B cell epitopes in PvRipr 3D structure. The protein chain is indicated by a gray and transparent (40%) surface. Epitopes B-PvRipr_(698-725)_, B-PvRipr_(751-771)_, B-PvRipr_(879-888)_, and B-PvRipr_(923-958)_ are highlighted in yellow, green, pink and blue, respectively. **(A)** and **(B)** show different rotations of the protein.

Using full length PvRipr, the prediction of B cell linear epitopes present in the protein was performed. A total of four epitopes were predicted, all of them present in the C-terminal region of PvRipr (Ct-PvRipr). They were composed of 10 to 36 amino acids and named according to the position of the first and the last amino acid on the sequence as B-PvRipr_(698-725)_, B-PvRipr_(751-771)_, B-PvRipr_(879-888)_, and B-PvRipr_(923-958)._ Of all identified epitopes, B-PvRipr_(923-958)_ was the only sequence predicted in all algorithms used. The full amino acid sequence of each epitope and their length are shown in **Table 2**. All B cell linear epitopes predicted were exposed in the 3D structure of Ct-PvRipr as shown in **Figure 4**.

**Table 2:** Predicted B cell epitopes in PvRipr.

Epitope	Sequence	Length	Ellipro	Bcpreds	BepiPred 1.0	BepiPred 2.0	ABCpred	EMINI	LBtope	SVMtrip
B-PvRipr_(698-725)_	CVCSDSSQIEEGHLCVPKNKC KRKEYQQ	28	✓	✓	✓	✓	✓	–	✓	–
B-PvRipr_(751-771)_	FKRNERGICIPVDYCKNVTCK	21	✓	✓	–	✓	✓	–	✓	–
B-PvRipr_(879-888)_	KSRSGDSPEG	10	✓	–	✓	✓	✓	✓	✓	–
B-PvRipr_(923-958)_	GENYRPRGKDSPTGQAVKRGE ATKRGDAGQPGQAHS	36	✓	✓	✓	✓	✓	✓	✓	✓

All the 8 prediction algorithms used are listed in the table and checkmarks indicate that a respective epitope was either partially or completely predicted by the corresponding algorithm.

### 3.5 Confirmation of B cell linear epitopes in PvRipr

**Figure 5:**
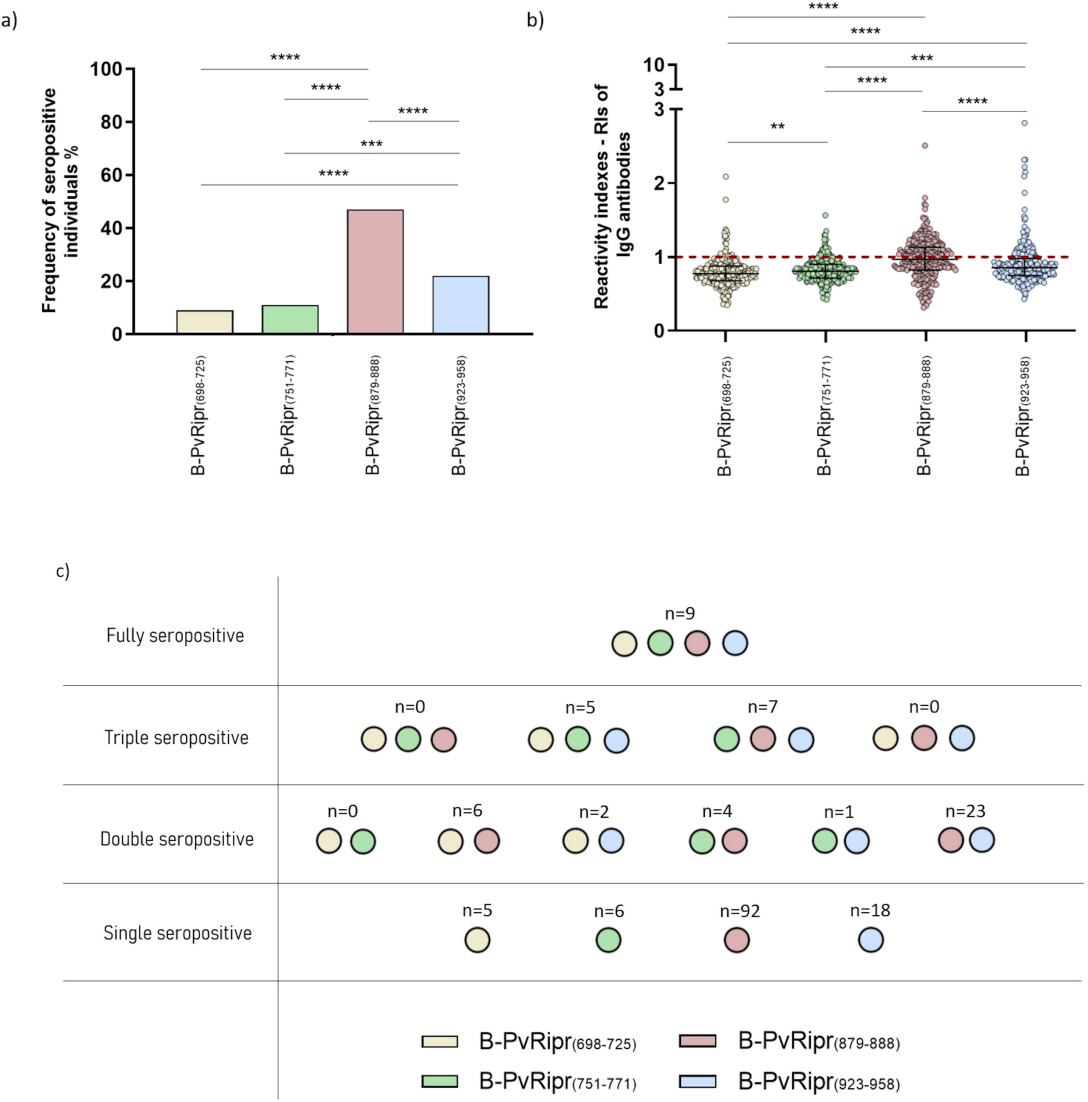
**(A)** Frequencies of seropositive individuals for IgG antibodies specific for B cell epitopes of PvRipr. The p values are p=0.0004 (***) and p<0.0001 (****), **(B)** RIs of IgG antibodies against B cell epitopes of PvRipr. The p values are p=0.0046 (**), p=0.0002 (***) and p<0.0001 (****) and **(C)** Different epitope response combinations identified in the studied population. Colored circles under n values indicate that the number of samples described is capable of recognizing epitopes of each respective color, as demonstrated in the colored rectangles.

ELISA assays were performed with four synthetic peptides corresponding to each one of the predicted B cell epitopes and plasma samples of all the study population (n=300), in order to determine RIs of specific-epitope IgG antibodies. Observed frequencies were, 27 seropositive individuals for B-PvRipr_(698-725)_ (9%), 32 seropositive individuals for B-PvRipr_(751-771)_ (11%), 141 seropositive individuals for B-PvRipr_(879-888)_ (47%), and 65 seropositive individuals for B-PvRipr_(923-958)_ (22%). The frequency of seropositive individuals for B-PvRipr_(879-888)_ was higher than all other B-cell epitopes tested (p<0.0001), besides, the frequency of seropositive individuals to B-PvRipr_(923-958)_ was also higher in comparison to B-PvRipr_(698-725)_ and B-PvRipr_(751-771)_ (p<0.0001 and 0.0004, respectively), as demonstrated in **Figure 5a** (frequencies were compared using Fisher’s exact test). RIs against B-PvRipr_(698-725)_ ranged from 0.348 to 2.089 with a median of 0.774, B-PvRipr_(751-771)_ ranged from 0.422 to 1.564 with a median of 0.806, B-PvRipr_(879-888)_ ranged from 0.314 to 2.508 with a median of 0.970, and B-PvRipr_(923-958)_ ranged from 0.431 to 2.816 with a median of 0.855. As well as for frequencies, RIs of IgG antibodies against B-PvRipr_(879-888)_ were higher than all other peptides (P<0.0001), followed by RIs of B-PvRipr_(923-958)_ that were higher in comparison to B-PvRipr_(698-725)_ and B-PvRipr_(751-771)_ (p<0.0001 and 0.0002, respectively). Finally, RIs of B-PvRipr_(751-771)_ were higher compared to RIs against B-PvRipr_(698-725)_ (p=0.0046) (**Figure 5b**; RIs were compared using Mann-Whitney test).

Of 300 individuals, 178 (59%) responded to at least one of the B cell epitopes tested. Inside this group, only nine plasma samples (5.1%) were characterized as seropositive for all the peptides (fully seropositive individuals), while 12 individuals (6.7%) could recognize three different peptides (triple seropositive individuals). A higher frequency was observed for seropositive individuals for two peptides (double seropositive individuals) (n=36, 20.2%) (p<0.0001 in comparison to full seropositive individuals and p=0.0004 in comparison to triple seropositive individuals). Finally, most individuals could only recognize one of all predicted B-cell epitopes (n=121, 68%) (p<0.0001), as demonstrated in **Figure 5c** (frequencies were compared between groups using Fisher’s exact test).

Frequencies and RIs of IgG antibodies specific for each epitope were also compared inside the group of IgG seropositive individuals for recombinant PvRipr (n=131) as demonstrated in **Table 3**. In this group, the frequency profile remained similar to the one identified in the entire population: a higher frequency of seropositive individuals for B-PvRipr_(879-888)_ was also observed (n=70 - 53.4%) in comparison to all other tested epitopes (Fisher’s exact test, p<0.0001). Followed by the frequency of seropositive individuals to B-PvRipr_(923-958)_ (n=38 – 29%), which was higher in comparison to B-PvRipr_(698-725)_ (Fisher’s exact test, p<0.0001) and B-PvRipr_(751-771)_ (Fisher’s exact test, p=0.004). There were no statistical differences between frequencies of seropositive individuals for B-PvRipr_(698-725)_ (n=10 – 7.6%) and B-PvRipr_(751-771)_ (n=19 – 14.5%). On the other hand, RIs of B-PvRipr_(879-888)_ (median of 1.152) and B-PvRipr_(923-958)_ (median of 1.128) were subtly superior to epitope B-PvRipr_(751-771)_ (median of 1.083), but no to B-PvRipr_(698-725)_ (median of 1.113).

**Table 3:** Frequencies and RIs of epitope seropositive individuals in the IgG seropositive group for recombinant PvRipr .

	B-PvRipr_(698-725)_	B-PvRipr_(751-771)_	B-PvRipr_(879-888)_	B-PvRipr_(923-958)_
Seropositive	10 (7.6%) **** ^/^ ****	19 (14.5%) **** ^/^ **	70 (53.4%)	38 (29%) ****
Seronegative	121 (92.4%)	112 (85.5%)	61 (46.6%)	93 (71%)
Chi-square	ns	ns	p<0.0001 ****	p<0.0001 **** p=0.004 **
Seropositive	1.113 (1.029–1.285)	1.083 (1.011-1.125) * ^/^ *^/^	1.152 (1.047-1.245)	1.128 (1.043-1.291)
Seronegative	0.786 (0.703–0.870)	0.817 (0.747-0.897)	0.862 (0.747-0.914)	0.852 (0.776-0.900)
Mann Whitney	ns	ns	p=0.0159 *	p=0.022 *

Frequencies are listed as n values (%) and compared by Chi-square, while RIs are listed as median (IQR) and compared by Mann Whitney. Pink asterisks indicate p values to B-PvRipr_(879-888)_ and blue asterisks to B-PvRipr_(923-958)._ * = p < 0.05 / ** = p < 0.01 and **** = p < 0.0001.

### 3.6 Humoral immune response against PvRipr and exposure/protection indicatives

All identified RIs of antibodies against recombinant PvRipr and its B cell epitopes were combined with epidemiological data collected from the study population. First, all individuals were divided into three groups, each one containing 100 patients, according to RIs of IgG antibodies against recombinant PvRipr (from lowest to highest value). Based on this, the first tertile corresponded to all humoral immune response information and epidemiological data of 100 individuals with RIs of IgG against PvRipr with values ≤ 0.812, followed by the second tertile with 100 individuals with RIs from 0.820 to 1.065, and the last tertile with values ≥ 1.069. Of all investigated parameters, significant differences were found in RIs across population tertiles were observed for IgM antibodies against recombinant PvRipr (p=0.010) and for RIs of IgG antibodies against epitopes PvRipr_(751-771)_ (p<0.0001), B-PvRipr_(879-888)_ (p=0.0003) and B-PvRipr_(923-958)_ (P<0.0001), all comparisons were performed using Kruskal-Wallis test. No statistical differences were found between tertiles for any of the epidemiological data verified (**Table 4**).

**Table 4:** Humoral immune response and epidemiological data of the studied population divided by tertile.

Parameter	First tertile low IgG RI ≤ 0.812	Second tertile medium IgG RI 0.820-1.065	Third tertile high IgG RI ≥ 1.069	KW test – p value
Number of values	100	100	100	-
Frequencies of infected/non-infected individuals	26% *P. vivax* 4% *P. falciparum* 70% non-infected	28% *P. vivax* 6% *P. falciparum* 66% non-infected	26% *P. vivax* 7% *P. falciparum* 67% non-infected	-
IgM (RIs) - Median (IQR)	0.763 (0.566-1.019)	0.803 (0.638-1.054)	0.878 (0.696-1.174)	0.010
PvRipr_(698-725)_ (RIs) - Median (IQR)	0.745 (0.647-0.838)	0.786 (0.688-0.879)	0.803 (0.706-0.889)	0.087
PvRipr_(751-771)_ (RIs) - Median (IQR)	0.760 (0.688-0.845)	0.811 (0.744-0.878)	0.849 (0.755-0.949)	<0.0001
PvRipr_(879-888)_ (RIs) - Median (IQR)	0.881 (0.690-1.065)	1.018 (0.850-1.181)	1.018 (0.870-1.166)	0.0003
PvRipr_(923-958)_ (RIs) - Median (IQR)	0.778 (0.689-0.895)	0.860 (0.749-0.931)	0.903 (0.811-1.059)	<0.0001
Age (years) - Median (IQR)	29 (20-47.3)	31 (20-44)	35 (23-49.8)	0.156
Time of residence in endemic area (years) - Median (IQR)	32 (19-47.3)	31 (20-46)	31 (22-46)	0.854
Time of residence in the present address (years) - Median (IQR)	22.5 (12.3-40.8)	20 (12-37)	22 (13.5-38)	0.844
Months since the last malaria episode – Median (IQR)	5 (0-24)	2 (0-12)	3 (0-12)	0.478
Number of malaria episodes in the last Year - Median (IQR)	0 (0-2)	1 (0-1)	0 (0-2)	0.886
Number of previous malaria episodes – Median (IQR)	7 (3-20)	10 (4-20)	6 (3-14.5)	0.134

Values are expressed as median (IQR). KW, Kruskal Wallis.

All correlations were computed using a non-parametric Spearman correlation matrix, allowing the assessment of relationships between RIs and epidemiological parameters, as well as among different antibody classes and epitopes. The analyses indicated an inverse correlation between RIs of IgM antibodies against PvRipr and number of previous malaria episodes (p = 0.003/r = - 0.179). Values of IgG2 antibodies against recombinant PvRipr correlated with both time of residence in endemic area and time of residence in the present address (p = 0.002/r = 0.282 and p = 0.028/r = 0.228). RIs of IgG antibodies against epitopes B-PvRipr_(698-725)_, B-PvRipr_(751-771)_ and B-PvRipr_(923-958)_ were all correlated with time of residence in endemic area (p = 0.003/r = 0.187; p = 0.0001/r = 0.241; p = 0.003/r = 0.186, respectively). RIs of epitopes B-PvRipr_(751-771)_ and B-PvRipr_(923-958)_ also correlated with time of residence in the present address (p = 0.015/r = 0.164 and p = 0.012/r = 0.169). An inverse correlation between RIs of PvRipr_(751-771)_ and the number of malaria episodes in the last year was identified (p = 0.006/r = - 0.165). Finally, RIs against epitope B-PvRipr_(879-888)_ correlated inversely with months since the last malaria episode (p < 0.0001 and r = - 0.244).

**Figure 6:**
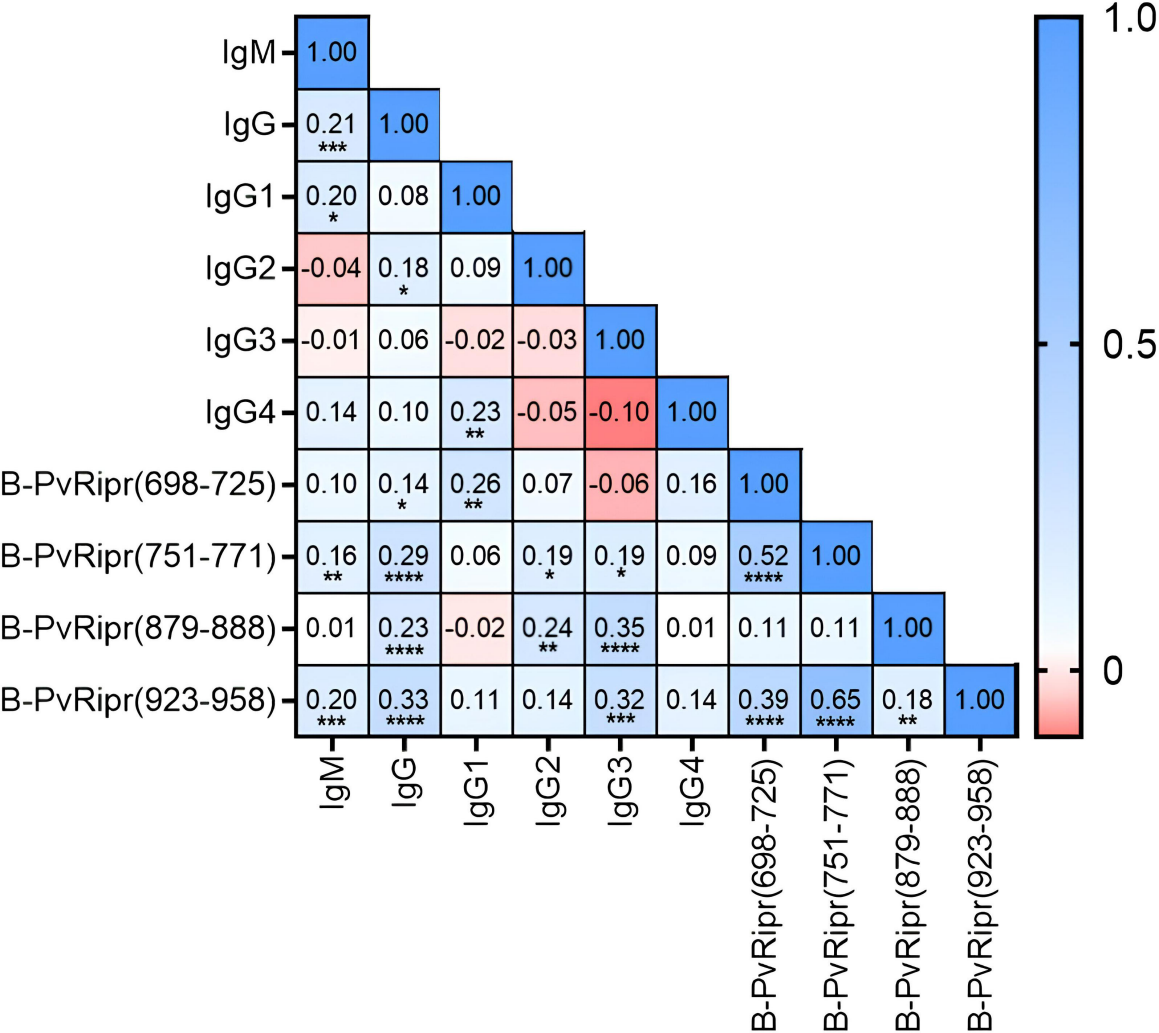
Correlation matrix of the humoral immune response against recombinant PvRipr and its B cell epitopes. Inside each box is the r value referring to the correlation between the antibodies that form the intersection. Asterisks were placed in boxes with significant p-values (* = p<0.05 / ** = p < 0.01 / *** = p < 0.001 and **** = p < 0.0001.

Correlations were also performed combining data found from all different antibodies against PvRipr and its epitopes. RIs of IgM antibodies against recombinant PvRipr presented correlation with IgG and IgG1 antibodies against PvRipr (p = 0.0002/r = 0.215 and p = 0.021/r = 0.202, respectively) and also against RIs of IgG antibodies against epitopes PvRipr_(751-771)_ and B-PvRipr_(923-958)_ (p = 0.007/r = 0.155 and p = 0.001/r = 0.198, respectively). Besides, RIs of IgG antibodies against PvRipr correlated with IgG2 (p = 0.045/r = 0.176) and with RIs of IgG antibodies against all tested epitopes (B-PvRipr_(698-725)_ – p = 0.016/r = 0.139; PvRipr_(751-771)_ – p < 0.0001/r = 0.287; B-PvRipr_(879-888)_ – p < 0.0001/r = 0.235; B-PvRipr_(923-958)_ – p < 0.0001/r = 0.331). RIs of IgG1 against recombinant PvRipr correlated with values of IgG4 and IgG antibodies against epitope B-PvRipr_(698-725)_ (p = 0.009/r = 0.230 and p = 0.003/r = 0.259, respectively). RIs of IgG2 against PvRipr correlated with RIs of IgG against epitopes PvRipr_(751-771)_ (p = 0.030/r = 0.190) and B-PvRipr_(879-888)_ (p = 0.007/r = 0.236). Finally, RIs of IgG3 antibodies against PvRipr correlated with IgG against epitopes PvRipr_(751-771)_ (p = 0.030/r = 0.189), B-PvRipr_(879-888)_ (p < 0.0001/r = 0.351), and B-PvRipr_(923-958)_ (p < 0.0001/r = 0.316) (**Figure 6**).

### 3.7 Depletion ELISA for epitopes B-PvRipr_(879-888)_ and B-PvRipr_(923-958)_

**Figure 7:**
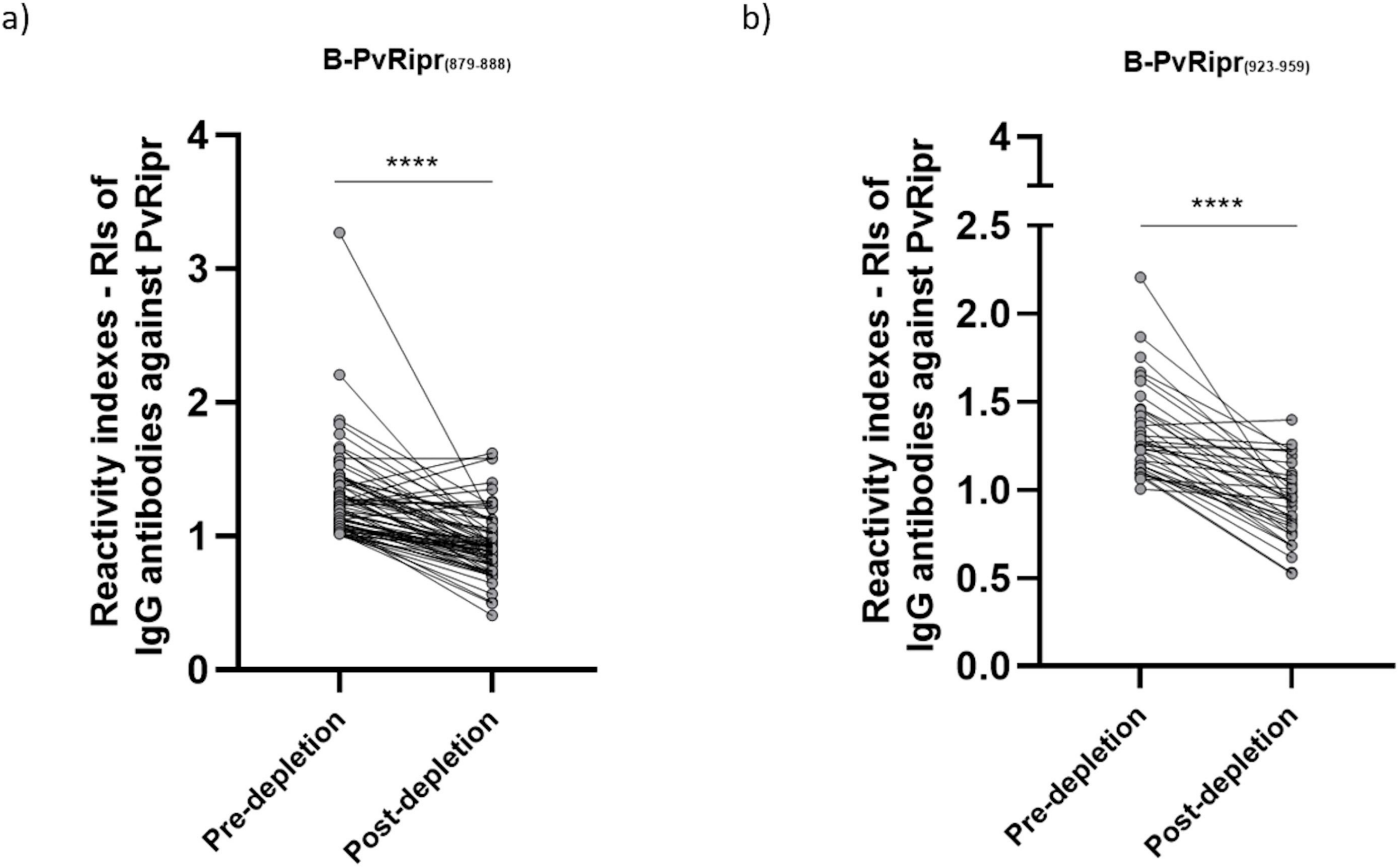
RIs of IgG antibodies against recombinant PvRipr pre and post-depletion of epitope-specific antibodies. Each grey circle represents the RI of an individual sample tested against recombinant PvRipr **(A)** RIs of 70 individuals seropositive for both PvRipr and B-PvRipr_(879-888)_. **(B)** RIs of 37 individuals seropositive for both PvRipr and B-PvRipr_(923-958)._ In both graphs **** indicates p < 0.0001.

The B-PvRipr_(879–888)_ and B-PvRipr_(923–958)_ epitopes were selected, and specific antibody responses against these sequences were depleted from the IgG response to recombinant PvRipr using an ELISA-based depletion assay. Individuals seropositive for IgG antibodies against both B-PvRipr_(879–888)_ and recombinant PvRipr (n = 70) showed a significant reduction in IgG RIs against PvRipr after depletion (p < 0.0001; pre-depletion: median 1.228, IQR 1.080–1.432; post-depletion: median 0.914, IQR 0.797–1.043) (**Figure 7a**). Similarly, individuals seropositive for IgG antibodies against both B-PvRipr_(923–958)_ and recombinant PvRipr (n = 37) also exhibited significantly lower IgG RIs after depletion (Mann-Whitney test, p < 0.0001; pre-depletion: median 1.277, IQR 1.132–1.457; post-depletion: median 0.934, IQR 0.775–1.070) (**Figure 7b**).

## 4 Discussion

The *P. vivax* merozoite is enveloped by a structurally complex protein coat, whose antigenic components have been extensively explored as vaccine candidates due to their capacity to elicit antibody-mediated immune responses capable of limiting erythrocyte invasion. While numerous surface and apical organelle proteins could potentially be targeted, the selection of an antigen for vaccine development requires careful consideration of factors such as structural and functional conservation, as well as the nature of the host immune response it induces. In this context, this study investigated full-length Ripr protein of *P. vivax* and the profile of the naturally acquired humoral immune response against this antigen and its most promising mapped epitopes in a population naturally exposed to malaria in the Brazilian Amazon.

The obtained results indicated that PvRipr is naturally immunogenic in the study population, as more than 60% of individuals presented either IgM or IgG antibodies against the recombinant protein. Due to the scarcity of studies investigating the naturally acquired immune response against Ripr even in *P. falciparum*, there are few results available in the scientific literature to compare with the findings of the present study. A study conducted by França and colleagues, used Ripr of *P. vivax* in ELISA assays with 144 samples of individuals from Solomon Island (48 children, 48 adolescents and 48 adults). Seropositivity rates increased with age, with 85% in children, 89% in adolescents, and 93% in adults (23). These frequencies are higher than those observed in our population, in which 43.7% of adult individuals were IgG seropositive. This difference may be related to the distinct malaria transmission profiles between the study sites. In addition to differences in malaria transmission intensity, this discrepancy may also be influenced by differences in sample size between the studies, particularly the substantially larger adult population analyzed in the present study (n=300). In another study, Rosado and colleagues used multiplex with a panel of 34 proteins of *P. vivax* to determine serological exposure markers, and demonstrated in a cohort of Peruvian individuals that IgG antibodies against PvRipr were correlated with both age and with antibodies against other invasion-related proteins, such as PvDBP, PvCyRPA, PvEBP and PvRBP (24). In *P. falciparum*, a cohort of Papuan New Guinean children with more than 9 years old, presented IgG antibodies in 50% of the population against recombinant PfRipr (only comprising amino acids 238-368) (25). Regarding the IgM antibody-mediated response, we found no reports of anti-Ripr IgM antibodies for *P. vivax* (neither for *P. falciparum*). Nevertheless, in our population approximately 35% of individuals were characterized as seropositive individuals for IgM against recombinant PvRipr. Even though we do not yet know the role of these specific antibodies against Ripr, it is important to highlight that IgM antibodies specific for other blood stage proteins have been described as multifunctional and able to act together with IgG antibodies in the activation of different branches of the immune system (26, 27). Although there is a scarcity of specific studies involving the PvRipr protein to compare the observed immune response profile, we can compare this response with other blood stage antigens of *P. vivax*, for which the naturally acquired immune response has already been investigated in Brazilian populations. Results from individuals residing in the Brazilian Amazon showed the existence of seropositive individuals to IgG antibodies in approximately half of the study populations used, as in the case of the PvDBP (49.5%), PvMSP1 (60%), PvRBP (60%), and PvMSP9 (74%) proteins (28–30).

In our population, a proportion of infected individuals (for both *P. vivax* and *P. falciparum*) were seronegative for IgG antibodies against recombinant PvRipr. This phenomenon has been consistently reported for several *P. vivax* blood-stage antigens, such as RBP, GAMA, MSP-9, RALP1, and VIR (6, 31–33), indicating that seronegativity among infected individuals is a common feature of malaria-endemic populations. Several factors may contribute to this observation, including differences in prior exposure history, transmission intensity in respective endemic areas, duration of infection, host genetic background, and the highly complex and heterogeneous nature of the immune response against *Plasmodium* parasites.

With respect to IgG subclasses, higher frequencies of seropositive individuals were observed for IgG1 and IgG2 antibodies, followed by IgG3, and finally IgG4. In relation to RIs, IgG2 presented higher levels in comparison to all other subclasses, followed by IgG1, IgG3, and IgG4. Comparing our results with the data obtained in the same study performed using the cohort of 206 Papua New Guinean children and 24 adults, plasma samples were investigated in ELISA assays against recombinant PfRipr, and authors demonstrated exclusively frequencies of seropositive individuals for IgG1 (47%) and IgG3 (62.1%) in children and frequencies of all subclasses in adults: IgG1 (63%), IgG2 (13%), IgG3 (59%) and IgG4 (4.5%) (25). In fact, in our results similar frequencies were observed for IgG1 (64.1%) and IgG3 (49.6%). However, frequencies of IgG2 and IgG4 were significantly higher in our study (64.9% and 31.3%, respectively). Nevertheless, this can be expected due to significant differences in both works, such as the plasmodial species under study, the epidemiological profile of each population, and the large discrepancy in the number of adult participants. In relation to other blood stage antigens investigated in populations of the Brazilian Amazon, as in our work, the prevalence of IgG1 and IgG2 antibodies was also observed in relation to other subclasses, as demonstrated in studies with PvRBP (IgG1 - 86% and IgG2 – 39%) and PvMSP9 (IgG1 - 60% and IgG2 - 63%) (28, 30).

Traditionally, IgG1 and IgG3 subclasses have been largely described as protective antibodies in malaria, while IgG2 and IgG4 subclasses have been identified as pathogenic (34). IgG1 and IgG3 are cytophilic antibodies that act in the mechanism of antibody-dependent cellular inhibition (ADCI), binding to immune cells, such as monocytes, and collaborating with them in the inhibition of parasite growth. This mechanism is widely described in the literature and has already been associated with reduced risk of symptomatic malaria (35, 36). However, the complete role of all subclasses is still unclear when it comes to the complexity of malaria immunity, and there are still many conflicting questions about the influence of IgG2 and IgG4 antibodies. Researchers have demonstrated an age-dependent association between IgG2 antibodies against the blood stage protein Pf332 and protection from malaria in individuals from Daraweesh village, in Eastern Sudan (37). In another study, 283 malaria exposed individuals from Burkina Faso were monitored for one year, and obtained results through ELISA assays in association with logistic regression analysis indicated that IgG2 antibodies against epitopes of proteins ESA and MSP-2 were associated with low risk of infection (38). Finally, Dobaño and colleagues have demonstrated through both univariable and multivariable analysis that levels of IgG4 against pre-erythrocytic and erythrocytic *P. falciparum* antigens were potentially associated with immunity in African infants who were participants of a phase 3 trial with RTS,S vaccine. The obtained results suggested that the pattern of cytophilic and non-cytophilic IgG antibodies is much more complex than initially thought and that it might be antigen-dependent, with these two types of subclasses possibly involved in protection (39).

Concerning the specific B cell epitope response investigated, four linear epitopes were identified in PvRipr. From these, epitopes B-PvRipr_(879-888)_ and B-PvRipr_(923-958)_ presented not only higher frequencies of seropositive individuals but also higher RIs in comparison to the other tested epitopes. Interestingly, considering multiple-epitope seropositive individuals (all individuals who are able to recognize more than one epitope), all epitope combinations found include at least the recognition of one of these most promising epitopes cited. In other words, there is no individual in this population that presents IgG antibodies against multiple epitopes without IgG antibodies against epitopes B-PvRipr_(879-888)_ and/or B-PvRipr_(923-958)._ An important observation is that not only B-PvRipr_(879-888)_ but also B-PvRipr_(923-958)_ were predicted by EMINI surface accessibility, a tool that calculates the probability of a determined epitope being exposed on the surface of a given antigen. None of the other epitopes were predicted by this algorithm. The presence of B-PvRipr_(879-888)_ and B-PvRipr_(923-958)_ on the surface of PvRipr, perhaps, explains higher frequencies of seropositive individuals and higher RIs observed for these two epitopes. Based on these observations, we used the epitopes B-PvRipr_(879–888)_ and B-PvRipr_(923–958)_ in depletion ELISA assays and demonstrated that IgG antibodies specific to these sequences directly contribute to RIs against recombinant PvRipr, reinforcing the immunodominant nature of these epitopes.

Blood stages of *Plasmodium* are responsible for malaria symptoms, especially the invasion and rupture of erythrocytes, a phase of the cycle characterized by extensive stimulation of the immune system. Considering that clinical immunity depends on successive exposures to *Plasmodium* over years of residence in endemic areas, epidemiological parameters of study populations are important tools for a better understanding of the observed immune response. Our results indicated that RIs of IgG2 and IgG antibodies (specific for epitopes B-PvRipr_(698-725)_, B-PvRipr_(751-771)_ and B-PvRipr_(923-958)_) were correlated with the time of residence in endemic area. In other words, this means that individuals who have resided in this region for a longer period, some for their entire lives, have possibly been exposed to *Plasmodium* numerous times and exhibit higher levels of antibodies against specific regions of PvRipr. These findings are consistent with studies of naturally acquired immunity against other blood-stage antigens of *P. vivax*. It is important to highlight that even the presence of high levels of antibodies specific to PvRipr or its epitopes does not mean that these antibodies are in fact protective. To confirm the possible protective role of these antibodies, functional assays such as growth inhibition assays (GIA) or opsonization tests would be necessary. Despite these findings, the obtained results did not indicate strong correlations between exposure or protection indicatives. Some possible explanations for the lack of associations may involve biological factors, such as the variability of each individual’s immune response, or methodological factors, such as the accuracy of the data provided by patients during the interview, one of the limitations of our study.

In a study conducted by Healer and colleagues, a GIA was performed with *P. falciparum* trophozoites and monoclonal antibodies against different proteins of the complex Rh5/CyRPA/Ripr, tested alone and combined. The results of the paper demonstrated that antibodies against C-terminal region of PfRipr (Ct-Ripr - comprising amino acids 604-1086) induced higher inhibition levels than all other antigens separated and even against full-length PfRipr (40). Even though the cited work involves another plasmodial species, it is important to highlight that Ct-Ripr of *P. falciparum* has 48.28% homology with Ripr protein of *P. vivax*. Interestingly, all B cell epitopes predicted in the present study (including B-PvRipr_(879-888)_ and B-PvRipr_(923-958)_) are inserted in this same portion of the protein, the C-terminal region, described as the one responsible for the induction of potent neutralizing antibodies, capable of blocking the formation of the erythrocyte invasion complex. Although specific antibody levels for these two sequences correlate with different epidemiological parameters and even with the RIs of other antibodies, it is important to emphasize that most of these correlations are modest, with low biological value (r < 0.3). Despite this, antibodies against B-PvRipr_(923-958)_ were strongly correlated with IgG antibodies against PvRipr and antibodies against both B-PvRipr_(879-888)_ and B-PvRipr_(923-958)_ were strongly correlated with IgG3 antibodies against recombinant PvRipr. This result was surprising, since frequency and RIs of IgG3 antibodies observed were relatively low compared to the other subclasses investigated in the response against PvRipr. Even though we cannot assume that the IgG3 antibodies identified in our population are protective, it is important to highlight that IgG3 antibodies have been widely cited in the scientific literature for their protective role, including studies investigating other blood stage antigens, such as MSP1, MSP2, MSP3, and GLURP (41–44). IgG3 is a cytophilic antibody subclass widely recognized as a fundamental part of the protective immune response against malaria, being described as the most potent activator of complement, besides, a mediator of opsonic phagocytosis and ADCI (45–47). Following this line of reasoning, we can suggest that these two specific epitopes may play an important role in the immune response to malaria by possibly inducing protective IgG3 antibodies and contributing directly to the acquisition of clinical immunity. Again, this hypothesis can only be confirmed through studies using functional assays.

During this study, the recombinant PvRipr used in the antibody assays was expressed in *E. coli*, recovered from inclusion bodies, and purified under denaturing conditions, without a subsequent refolding step. Under these circumstances, the protein is expected to populate a spectrum of non-native structural states, including partially unfolded or compact misfolded conformations, rather than its native disulfide-stabilized architecture. Consequently, the ELISA measurements predominantly capture IgG responses directed against epitopes that remain exposed or structurally accessible within these non-native forms. Although we did not perform structural validation assays such as circular dichroism, native PAGE, or parasite-based immunodetection, this methodological framework is consistent with numerous immunoepidemiological studies that have characterized naturally acquired antibody responses to merozoite antigens expressed in *E. coli* without *in vitro* refolding (6, 28, 30, 48–54). In such contexts, recognition of the recombinant antigen by naturally exposed individuals has been widely applied as a proxy of antigenicity and as evidence of the presence of immunologically relevant linear or quasi-linear determinants, even in the absence of preserved conformational epitopes. Similarly, the synthetic peptides employed in this work were selected through linear B-cell epitope prediction tools and were therefore synthesized in fully reduced form, without disulfide bond formation even when cysteine residues were present. This approach, which is consistent with the linear nature of the prediction algorithms, intentionally avoided mimicking the native EGF-like cysteine-stabilized domains of PvRipr. Together, these methodological aspects indicate that the responses measured here do not account for conformational epitopes and may explain the comparatively low reactivity observed for cysteine-rich peptides. In line with these methodological considerations, the differences in antibody recognition between cysteine-containing and cysteine-free peptides can be largely explained by their structural characteristics. Because the cysteine-rich peptides were synthesized as linear, fully reduced sequences, they did not reproduce the disulfide-stabilized EGF-like domains that characterize the native C-terminal region of PvRipr. This lack of native folding, together with their intrinsically low predicted surface accessibility, likely contributed to the markedly lower IgG reactivity observed for peptides B-PvRipr _(698–725)_ and B-PvRipr_(751–771)_. In contrast, the cysteine-free epitopes B-PvRipr_(879–888)_ and B-PvRipr_(923–958)_ were consistently identified by multiple prediction algorithms, including surface accessibility analyses, as regions more exposed on the native protein surface. These peptides also demonstrated the highest responder frequencies, the strongest antibody reactivity indexes, and robust correlations with total IgG and IgG3 specific to PvRipr, supporting their biological relevance as accessible and immunodominant linear epitopes.

As previously described in *P. falciparum*, Ripr acts in a complex with Rh5 and CyRPA, and the entire complex has already been investigated in vaccine prototypes for this species (55). Once *P. vivax* lacks Rh5 (19), PvCyRPA could be a promising antigen to be associated with PvRipr. In a study using a library of 38 P*. vivax* antigens tested in association with samples from a cohort of 264 Papua New Guinean children, PvCyRPA was highlighted as a promising vaccine candidate by presenting one of the best results in relation to the algorithm used to identify the protective potential of each investigated protein, even with low antibody levels induced (56). Recently, our research group has described the humoral and cellular immune response against recombinant PvCyRPA and its B and T CD4 cell epitopes in 90 individuals from the Brazilian Amazon. The results demonstrated that PvCyRPA is naturally immunogenic in this population and contains a promising region with overlapped epitopes, which might be capable of inducing both humoral and cellular immune responses (7). In this context, previous works have already indicated a synergistic role of CyRPA and Ripr in *P. falciparum.* One of them was conducted using GIA with *P. falciparum* trophozoites and combinations of monoclonal antibodies against proteins of the complex Rh5/CyRPA/Ripr. Performed assays indicated that the association of monoclonal antibodies against PfCyRPA and PfRipr had the highest inhibition level in comparison to not only all other combinations tested but also against each protein of the complex individually (40). In another study, Bliss’ and Loewe’s models were used to characterize additive and synergistic effects of different blood stage malaria vaccine candidates in *P. falciparum* GIA, and again, a synergistic effect was observed when antibodies against PfCyRPA and PfRipr were combined (57). Together, these findings only reinforce how promising the combination of these blood-stage antigens in chimeric vaccines can be. Finally, it is also important to highlight that B-PvRipr_(879-888)_ localization is compatible with the recent study of Williams and colleagues, where growth-inhibitory antibody epitopes of *P. falciparum* Ripr, inserted in the C-terminal EGF like domains, specifically PfRipr EGF (7–8) (comprising amino acids 817-879), were fused to full-length PfCyRPA and improved the level of *in vitro* parasite growth inhibition, advancing as the vaccine candidate RH5.1 + R78C/Matrix-M™ to phase 1 clinical trial (58). However, despite these promising results, these antigens have not yet been investigated in vaccine constructs for *P. vivax.* In summary, the results presented in this paper demonstrate the characteristics of the humoral immune response against full-length PvRipr in individuals naturally exposed to malaria and indicate the existence of promising regions in this merozoite antigen that can be investigated and used in future studies.

## 5 Conclusion

This study provides the characterization of the naturally acquired humoral immune response against full-length PvRipr in individuals exposed to malaria in the Brazilian Amazon. PvRipr was shown to be naturally immunogenic, with more than half of the studied population presenting IgM or IgG antibodies against the protein. Among the predicted epitopes, B-PvRipr_(879-888)_ and B-PvRipr_(923-958)_ stood out as the most immunoreactive, showing strong correlations with cytophilic IgG3 antibodies, a subclass often associated with protection in malaria. However, no clear association was observed between antibody responses to PvRipr and epidemiological markers of exposure or immunity, suggesting that the functional role of these antibodies in protection remains unclear. These results highlight the immunogenic regions of PvRipr but indicate that further studies, including functional assays, are required to determine whether antibodies elicited by PvRipr can mediate inhibitory or protective mechanisms. Overall, this work provides a detailed description of the humoral response to PvRipr in naturally exposed individuals and identifies specific epitopes that could be considered in future studies exploring immune mechanisms or multi-antigen vaccine strategies, while acknowledging that the protective relevance of these responses has not yet been established.

## Data Availability

The datasets presented in this study can be found in online repositories. The names of the repository/repositories and accession number(s) can be found in the article/supplementary material.
